# Mitochondrial genomes of genus *Atta* (Formicidae: Myrmicinae) reveal high gene organization and giant intergenic spacers

**DOI:** 10.1590/1678-4685-GMB-2018-0055

**Published:** 2020-01-13

**Authors:** Josefa T. V. Barbosa, Marcílio S. Barbosa, Suzyane Morais, Antônio E. G. Santana, Cicero Almeida

**Affiliations:** 1 Laboratory of Genetics Resources, Campus Arapiraca, Universidade Federal de Alagoas, Arapiraca, AL, Brazil.; 2 Centro de Ciências Agrárias, Universidade Federal de Alagoas, Arapiraca, AL, Brazil.

**Keywords:** Ants, evolution, mitogenomes

## Abstract

The ants of the genus *Atta* are considered important pests to agriculture in the Americas, although *Atta* species are also important contributors to ecosystem functions in the various habitats in which they occur. The aim of this study was to assemble four complete mitochondrial genomes of the genus *Atta*, construct the phylogenomic tree, and analyze the gene content, order, and organization. The mitogenomes of *A. colombica*, *A. opaciceps*, *A. texana*, and *A. sexdens rubropilosa* comprise 18,392, 19,257, 19,709, and 19,748 bp, respectively. The four Atta mitogenomes showed the charactistics typical of those of insects, with 13 protein-coding genes, 22 tRNAs, and 2 rRNAs, with genes displayed in the conventional order. Analysis for intergenic spacer regions showed that *Atta* intergenic spacers are larger than those of the outgroups. Phylogenomic analyses using partial cytochrome oxidase I gene sequences showed similar topologies to previous phylogenetic analyses, with high clade support values. We conclude that *Atta* mitogenomes are characterized by high conservation in gene order and have giant intergenic spacers in the genus *Atta*.

The ants of the genus *Atta* are leafcutters belonging to the tribe Attini (Hymenoptera: Formicidae: Myrmicinae) and are considered important pests to agriculture in the Americas, although *Atta* species are also important contributors to ecosystem functions in the various habitats in which they occur. The species widespread in Brazil are *A. bisphaerica* Forel, 1908, *A. capiguara* Gonçalves, 1944, *A. cephalotes* Lineu, 1758, *A. goiana* Gonçalves, 1942, *A. laevigata* F. Smith, 1858, *A. opaciceps* Borgmeier, 1939, *A. robusta* Borgmeier, 1939, *A. sexdens piriventris* Santschi, 1919, *A. sexdens rubropilosa* Forel, 1908, *A. sexdens sexdens* Lineu, 1758, *A. silvai* Gonçalves, 1982, and *A. vollenweideri* Forel, 1939.

Phylogenetic analyses using gene fragments of cytochrome oxidase I, tRNA leucine, and cytochrome oxidase II revealed four clades: (1) *A. texana*, *A. mexicana*, and *A. insularis* in the Archeatta clade; (2) *A. colombica* and *A. cephalotes* in the Atta s. str. clade; (3) *A. opaciceps*, *A. laevigata*, *A. capiguara*, *A. bisphaerica*, *A. vollenweideri* Forel 1939 and *A. saltensis* in the Epiatta clade, and (4) *A. sexdens* and *A. robusta* in the Neoatta clade (Bacci *et al.*, 2009). These phylogenetic relationships showed some clades with low branch support, and the phylogenetic analysis using complete mitogenomes provided robust inferences. Complete mitogenomes allow the analysis of rearrangements, deletions, duplications, and inversions among mitogenomes. However, complete mitogenomes for the genus *Atta* have been described only for *A. laevigata* (Rodovalho *et al.*, 2014) and *A. cephalotes* (Suen *et al.*, 2011). For other species of the subfamily Myrmicinae, mitogenomes are available for *Pristomyrmex punctatus* (Hasegawa *et al.*, 2011), three species of *Solenopsis* (Gotzek *et al.*, 2010), *Vollenhovia emeryi* (Liu *et al.*, 2016), *Wasmannia auropunctata* (Duan *et al.*, 2016), and *Myrmica scabrinodis* (Babbuci *et al.*, 2014).

In this study, four complete mitochondrial genomes of the genus *Atta* were assembled. Mitogenomes were utilized for phylogenomic analyses, gene content, and order for exploring the evolution of the genus *Atta.* For development of mitogenomes, reads of *A. opaciceps* were sequenced, and reads of *A. colombica*, *A. texana*, and *A. sexdens rubropilosa* were downloaded from the NCBI database and utilized to assemble the complete mitogenomes.

For *A. opaciceps*, the biological sample was collected in the state of Alagoas, Brazil, and DNA extraction was performed using the cetyltrimethylammonium bromide (CTAB) extraction method (Doyle and Doyle, 1987). The quality and quantity of the extracted DNA were verified by visualization on a 1% agarose gel and spectrophotometryer, respectively. The DNA sample was fragmented by sonication into 500–600 bp to construct the sequencing library, and fragments were ligated with adapters using the Nextera DNA Sample Preparation” (Illumina) kit. Sequencing of paired-end fragments with a size of 100 nt was done on a Illumina HiSeq2500 platform at the Central Laboratory for High Performance Technologies in Life Sciences (LacTad) at the State University of Campinas (UNICAMP) in Campinas, São Paulo.

For *A. colombica* (SRR3187022 and SRR3168931), *A. texana* (SRR5438011), and *A. sexdens rubropilosa* (SRR5651498), short reads were obtained from public data in NCBI, from which the SRA files were unpacked into FASTQ using the FASTQ-DUMP tool executable from the SRA Toolkit. FASTQ files were then filtered with a minimum quality of 10, converted into FASTA files, and utilized for genome assembly. Thirty million reads of *A. colombica*, 24 million reads of *A. opaciceps*, 3.3 million reads of *A. texana*, and 4.2 million reads for *A sexdens rubropilosa* were used.

To obtain the mitochondrial genome of the four species, reads were mapped using the mitochondrial genome of *A. laevigata* as reference, using the software Geneious R9 (http://www.geneious.com). The draft mitogenomes were checked using contigs from the *de novo* assembling generated by Ray software (Boisvert *et al.*, 2012), performed using parameter kmer 31; the largest contig was analyzed using BLAST for mitochondrial identification. Genome annotation was achieved using the MITOS web server (Bernt *et al.*, 2013) and confirmed with Geneious software using the mitochondrial genome of *A. laevigata* as reference. The annotations were checked and, where necessary, manually corrected. A graphic representation of the mitochondrial genome of *A. opaciceps* was created using Geneious.

Six mitochondrial genomes for the genus Atta and other three genomes from the Myrmicinae subfamily were utilized for phylogenetic inferences (Table 1). The mitogenomes for A. *cephalotes*, *A. laevigata*, *M. scabrinodis*, *P. punctatus*, and *S. richteri* were obtained from the NCBI and the mitogenomes of the *A. colombica*, *A. opaciceps*, *A. texana*, and *A sexdens rubropilosa* were assembled in this study. The mitogenome sequences were aligned using the program MAFFT v7.017 (Katoh and Standley, 2013) implemented as the “Multiple align” tool in Geneious R9*, t*he evolutionary history was inferred using the maximum likelihood (ML) method based on the GTR+I+G nucleotide substitution model (Nei and Kumar, 2000), and branch support was assessed with 1,000 bootstrap replicates. The nucleotide substitution model and ML analyses were conducted in MEGA7 (Kumar *et al.*, 2016). The genetic relationships among species were also investigated through a principal component analysis (PCA) using the function*glPca*in *R packageadegenet (* Jombart and Ahmed, 2011) and Single sequence repeats (SSRs); microsatellites were identified using Phobos software (Mayer, 2010).

**Table 1:** Mitochondrial genome of the genus *Atta* and other species of the subfamily Myrmicinae utilized as the outgroup. The genome sizes in base pairs (bp) are shown for the genome and the coding and noncoding regions. The genome annotation for tRNA, rRNA, and protein-coding gene (genes) and the NCBI code for the genomes are presented

Species	Size (pb)			Annotations			NCBI	References
	Genome	coding	non-coding	tRNA	rRNA	Genes		
*M. scabrinodis*	15310	14655	738	21	2	13	LN607806	Babbucci *et al*.,2014
*P. punctatus*	16180	14693	1550	22	2	13	AB556947	Hasegawa *et al*.,2011
*S. richteri*	15560	14673	915	23	2	13	HQ215539	Gotzek *et al*.,2010
*A. cephalotes*	18815	14888	3946	23	2	13	HQ415764	Suen *et al*.,2011
*A. colombica*	18392	14756	3655	22	2	13	KY950644	This study
*A. laevigata*	18729	14684	3881	22	2	13	KC346251	Rodovalho *et al*.,2014
*A. opaciceps*	19257	14840	4433	22	2	13	KY9[50643	This study
*A. texana*	19709	14844	4880	22	2	13	MF417380	This study
*A. sexdens rubropilosa*	19748	14513	5235	22	2	13	MF591717	This study

The mitogenomes of *A. colombica*, *A. opaciceps*, *A. texana*, and *A. sexdens rubropilosa* contained 18,392, 19,257, 19,709, and 19,748 bp, respectively (Table 1). After obtaining the final mitogenomes, mapping with short reads was carried out, allowing to map the reads with no errors and 100% identity, which resulted in an average coverage of 139.2 x for *A. opaciceps*, 62.2 x for *A. colombica*, 304 x for *A. texana*, and 35.4 x for *A. sexdens rubropilosa* (Figure S1).

**Figure 1:**
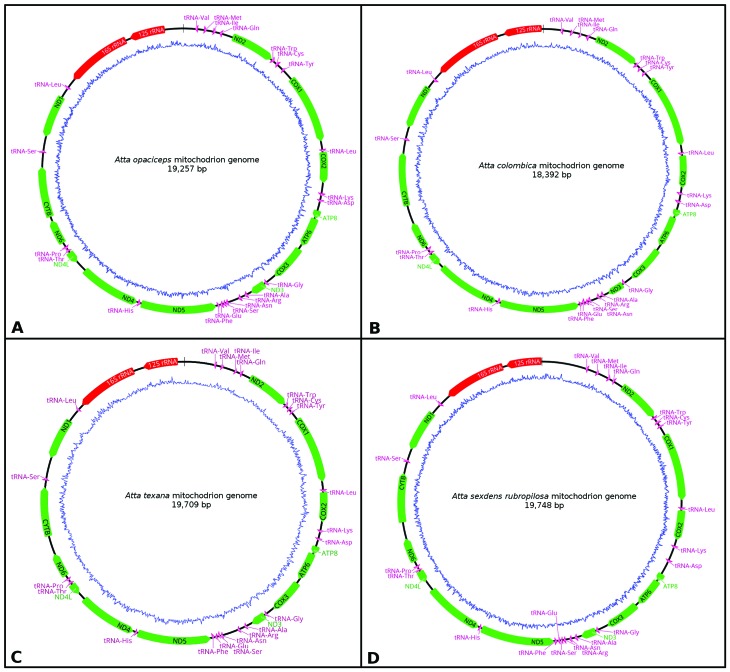
Complete gene map of *Atta opaciceps* (A), *Atta colombica* (B), *A. texana* (C), and *Atta sexdens rubropilosa* (D) mitogenomes. Genes in the circle and outside the circle are transcribed in clockwise and counterclockwise directions, respectively. The protein-coding genes are shown in green, rRNAs in red, and tRNAs in purple. The green ring represents the A+T contents and the blue ring shows C+G contents.

The four *Atta* mitogenomes showed the typical characteristics of those for insects, with 13 protein-coding genes, 22 tRNAs, and 2 rRNAs, as well as the noncoding region (Figure 1 and Table 1), with the genes displayed in the same order and orientation as in the hypothesized ancestral mitogenome (Figure S2). The mitogenome arrangement for the genus *Atta* was identical, wherein the protein-coding genes and the rRNAs displayed the same order and orientation. Additional tRNAs were observed between ATP8 and COX2 in *A. cephalotes* (Figure S2).

The A + T contents of mitogenomes were high, ranging from 72.7% (*A. sexdens rubropilosa*) to 82.5% (*A. texana*). For the coding region, the lowest A + T content was in COXI, COX3, and ATP6, whereas the highest A + T content was between ND5 and ND3. The A + T contents between the coding and noncoding regions were different, ranging from 77.6% (*A. cephalotes*) to 78.5% (*A. texana*), whereas the noncoding regions showed an A + T content ranging from 84.1% (*A. sexdens rubropilosa*) to 90.3% (*A. texana*). The four genomes of the genus *Atta* revealed 60 SSRs, which were evenly distributed, and the di-nucleotide motifs were more abundant, except for *A. cephalotes* for which the tetra-nucleotide motifs were more abundant (Figure S3).

The size of the whole non-coding (intergenic spacers) regions showed that *Atta* species have large intergenic spacers when compared with the outgroup (Figure 2A), ranging from 3,655 to 5,238 bp, whereas the outgroup showed spacers ranging from 738 to 1,550 bp (Table 1). The large intergenic spacers in the genus *Atta* are found in all intergenic spacers (Figure 2B). For the coding region, the sequences displayed similar length in the *Atta* and the outgroup (Table 1).

**Figure 2:**
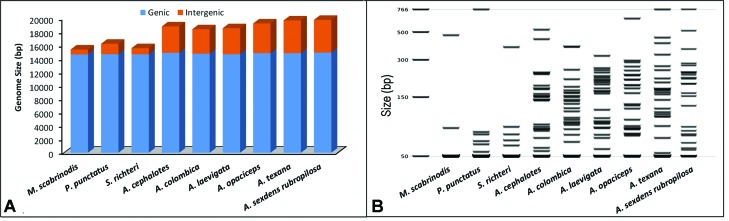
Genome size of the complete mitogenomes for *Atta* genus and outgroup. (A) Distribution of the genic and intergenic spacers. (B) Virtual gel showing the distribution of the intergenic spacers.

The phylogenetic analysis showed *A. texana* as the basal clade and the other species as the derived clade (Figure 3). The topologies obtained with the complete mitogenome (Figure 3A) and coding regions (Figure 3B) showed no difference. In both phylogenetic analyses, the branch-support values were high, with a bootstrap value of >96% for the *Atta* clades. The results of PCA for complete mitogenomes and the coding regions were different. PCA using complete genomes showed clear support for species delimitation in *Atta* (Figure S4A). When using only coding regions, *A. colombica* and *A. cephalotes* species formed one group and *A. laevigata* and *A. opaciceps* formed another (Figure S4B). In the PCA for complete mitogenomes, the first principal component separated *A. laevigata*, *A. opaciceps*, and *A. sexdens rubropilosa* from the other species, and the second principal component separated *A. colombica* and *A. cephalotes* from *A. texana*.

**Figure 3:**
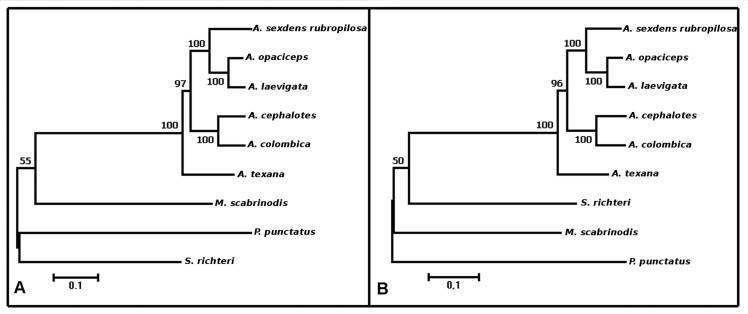
(A) Molecular phylogenetic analysis by Maximum Likelihood method for complete mitogenomes and (B) for coding regions. In both A and B, the supported values were estimated by bootstrap.

The nucleotide compositions in all analyzed *Atta* mitogenomes are characterized by a high frequency of A + T. The same result was reported previously for *A. laevigata* by Rodovalho *et al.* (2014), and the gene order and orientation are the same in all *Atta* mitogenomes, like in the ancestral insect mitochondrial genome (Cameron *et al.*, 2014). However, *Atta* mitogenomes were larger, suggesting a phylogenetic signal. The size variation is influenced by expansions in intergenic spaces, confirmed by the larger intergenic spacers found in *Atta* mitogenomes. Expansions in the intergenic spaces do not affect gene functions, and thus can be considered selectively neutral. Intergenic spaces from other insect mitogenomes have been reported to range from 216 bp in *Naupactus xanthographus* (Song *et al.*, 2010) to 5,654 bp in *Protaetia brevitarsis* (Kim *et al.*, 2014), suggesting that *Atta* mitogenomes are characterized by larger intergenic spacers.

Mitogenomes have an impact on insect genetics, as they are widely utilized for phylogenetic studies. Regarding the genus *Atta*, Bacci *et al.* (2009) utilized partial mitochondrial gene sequences (COI, tRNA leucine, and COII) for phylogenetic analysis. However, the advent of next-generation sequencing technologies has resulted in the complete sequencing of mitogenomes, allowing robust phylogenetic analyses. This approach allows a phylogenetic reconstruction using complete mitogenomes and coding or noncoding regions (intergenic spaces). In the present study, two phylogenetic analyses were conducted, using the complete genomes and only the coding regions. The result showed that phylogenetic analysis using the complete mitogenomes was more informative in both ML and PCA analyses than that using the coding regions only, as the rate of substitution in the complete mitogenome was larger than that in coding regions, and the principal component separated the *Atta* species*.*

Topologies using complete mitogenomes were similar to the phylogeny generated with partial COI-tRNA-COII sequences (Bacci *et al.*, 2009), revealing *A. texana* in the basal clade, *A. cephalotes* and *A. colombica* in the second clade, *A. laevigata* and *A. opaciceps* in the third clade, and *A. sexdens rubropilosa* in the fourth clade. However, bootstrap values using mitogenomes were larger than those for partial COI-tRNA-COII, indicating more robust phylogenetic inference with mitogenomes. We conclude that *Atta* mitogenomes are characterized by high conservation in gene order and organization and by giant intergenic spacers.

## Supplementary material

The following online material is available for this study:

Figure S1 Coverage of *Atta* genomes after mapping of short reads.

Figure S2 Organization of the *Atta* mitogenomes compared with that of outgroups.

Figure S3 Comparative*analysis*of*microsatellites*inthe *mitochondrial genomes*of *Atta*.

Figure S4 Principal component analysis (PCA) for five species of the *Atta* genus and three outgroup species.
