# Supplementary material for: Mitochondrial genomes of genus Atta (Formicidae: Myrmicinae) reveal high gene organization and giant intergenic spacers
Source: Genet Mol Biol. 2020 Jan 13;42(4):e20180055. doi: 10.1590/1678-4685-GMB-2018-0055 (PMC7197989; doi:10.1590/1678-4685-GMB-2018-0055)
Supplement: Supplementary file 1 [file 1415-4757-GMB-42-04-e20180055-suppl1.pdf]

Genetics and Molecular Biology, 42, 3 (2019)

Supplementary Material to "Mitochondrial genomes of genus Atta (Formicidae: Myrmicinae) reveal high gene organization and giant intergenic spacers"

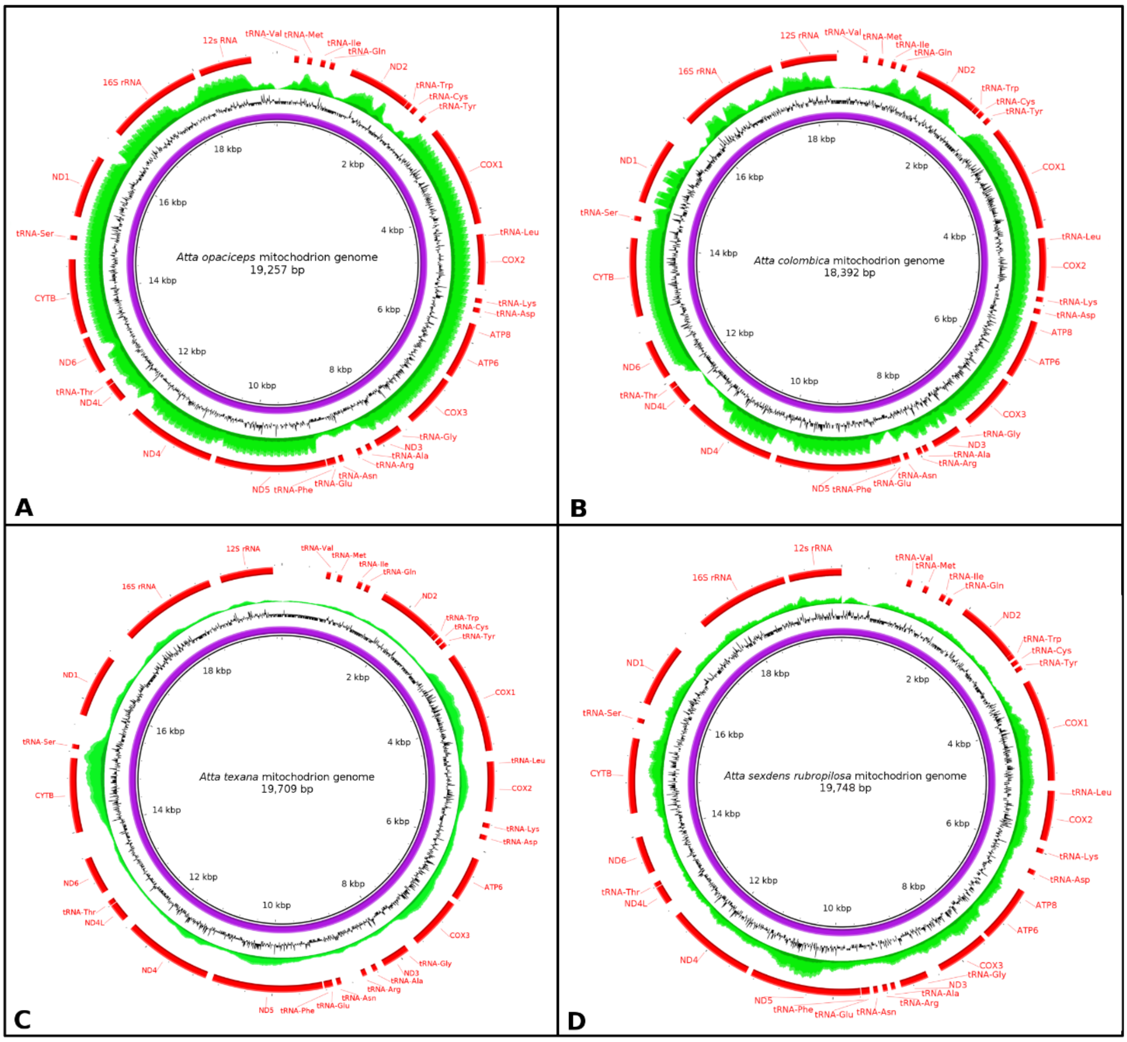

Figure S1 - Coverage of *Atta* genomes after mapping of short reads. The first ring represents the genome, the second represents CG content, and the third indicates coverage of the mapped reads. The annotations in red show the regions used for validation using sequencing following the Sanger method.
