# Supplementary material for: Mitochondrial genomes of genus Atta (Formicidae: Myrmicinae) reveal high gene organization and giant intergenic spacers
Source: Genet Mol Biol. 2020 Jan 13;42(4):e20180055. doi: 10.1590/1678-4685-GMB-2018-0055 (PMC7197989; doi:10.1590/1678-4685-GMB-2018-0055)
Supplement: Supplementary file 2 [file 1415-4757-GMB-42-04-e20180055-suppl2.pdf]

Supplementary Material to "Mitochondrial genomes of genus Atta (Formicidae: Myrmicinae) reveal high gene organization and giant intergenic spacers"

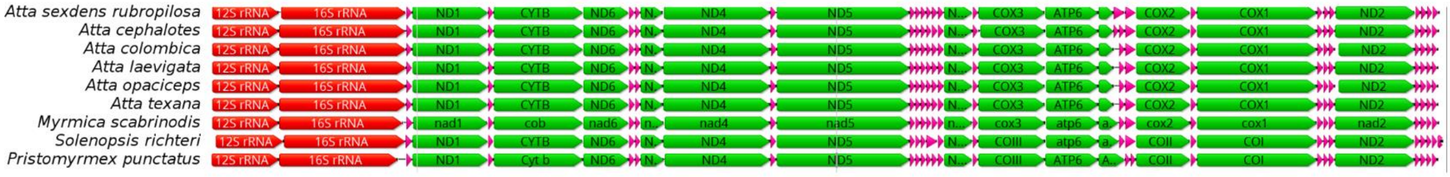

Figure S2 - Organization of the *Atta* mitogenomes compared with that of outgroups. All coding regions were displayed in the same order and direction.
