# Supplementary material for: Mitochondrial genomes of genus Atta (Formicidae: Myrmicinae) reveal high gene organization and giant intergenic spacers
Source: Genet Mol Biol. 2020 Jan 13;42(4):e20180055. doi: 10.1590/1678-4685-GMB-2018-0055 (PMC7197989; doi:10.1590/1678-4685-GMB-2018-0055)
Supplement: Figure S3 [file 1415-4757-GMB-42-04-e20180055-suppl3.pdf]

# Supplementary Material to "Mitochondrial genomes of genus Atta (Formicidae: Myrmicinae) reveal high gene organization and giant intergenic spacers"

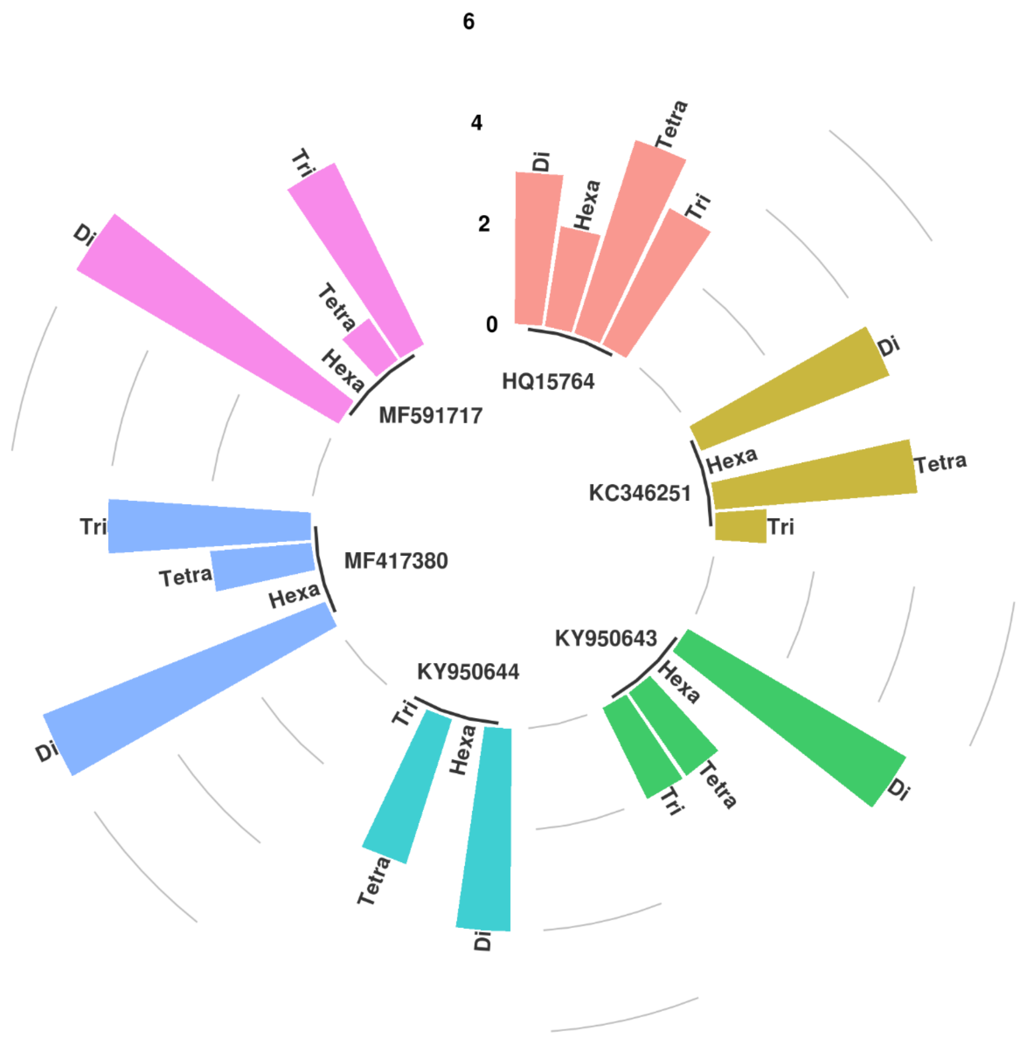


**Figure S3** - Comparative *analysis* of *microsatellites* in the *mitochondrial genomes* of *Atta*.
