# Supplementary material for: Mitochondrial genomes of genus Atta (Formicidae: Myrmicinae) reveal high gene organization and giant intergenic spacers
Source: Genet Mol Biol. 2020 Jan 13;42(4):e20180055. doi: 10.1590/1678-4685-GMB-2018-0055 (PMC7197989; doi:10.1590/1678-4685-GMB-2018-0055)
Supplement: Figure S4 [file 1415-4757-GMB-42-04-e20180055-suppl4.pdf]

Supplementary Material to "Mitochondrial genomes of genus Atta (Formicidae: Myrmicinae) reveal high gene organization and giant intergenic spacers"

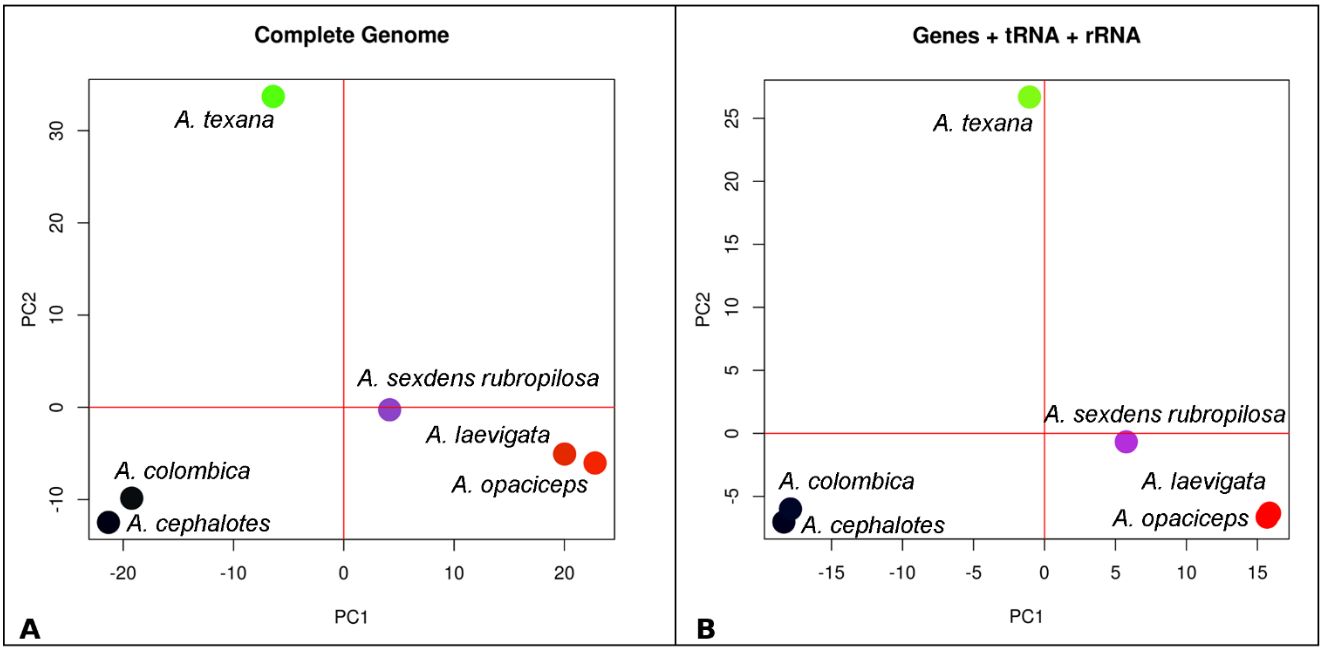

Figure S4 - Principal component analysis (PCA) for five species of the *Atta* genus and three outgroup species. (A) PCA using complete mitogenomes and (B) PCA using the coding regions (genes + tRNA + rRNA). The colors denote values for the first two principal component (PC) axes.
